# Berberine-induced ferroptosis as a novel anti-cancer strategy: Molecular, epigenetic and translational perspectives

**DOI:** 10.17179/excli2026-9385

**Published:** 2026-07-07

**Authors:** Neetu Agrawal, Gaurav Gupta, Surya Nath Pandey, A Rekha, Mano Priya Vijayan, Dinesh Kumar Chellappan, Keshav Raj Paudal, Sachin Kumar Singh, Kamal Dua

**Affiliations:** 1 Institute of Pharmaceutical Research, GLA University, Mathura, Uttar Pradesh, India; 2 Centre for Research Impact & Outcome, Chitkara College of Pharmacy, Chitkara University, Rajpura, Punjab 140401, India; 3 Department of Pharmacology, Teerthanker Mahaveer College of Pharmacy, Teerthanker Mahaveer University, Moradabad 244001 Uttar Pradesh, India; 4 Dr. D. Y. Patil Medical College, Hospital and Research Centre, Pimpri, Pune, India; 5 Faculty of Health Sciences, Villa College, Maldives; 6 Department of Life Sciences, School of Pharmacy, IMU University, Bukit Jalil, Kuala Lumpur 57000, Malaysia; 7 National Institute of Complementary Medicine Health Research Institute and School of Science, Western Sydney University, Westmead, NSW2145, Australia; 8 School of Pharmaceutical Sciences, Lovely Professional University, Phagwara, Punjab 144411, India

**Keywords:** berberine, ferroptosis, lipid peroxidation, GPX4, nanodelivery, chemosensitization, epigenetics, miRNA

## Abstract

Cancer cells frequently evade therapies that depend on apoptosis, necessitating the exploration of alternative cell death mechanisms. Ferroptosis, an iron-dependent regulated cell death characterized by lethal lipid peroxidation, has emerged as a promising strategy for cancer treatment. Recent studies have identified berberine, an isoquinoline alkaloid derived from Coptis chinensis and Berberis species, as an inducer of ferroptosis in various malignancies through its multitarget effects. This review systematically elucidates the molecular pathways through which berberine induces ferroptosis. These pathways include the inhibition of the System Xc^−^/glutathione/glutathione peroxidase 4 antioxidant axis, disruption of iron homeostasis via ferritinophagy, inhibition of mitochondrial complex I, and regulation of the upstream regulators p53, nuclear factor erythroid 2-related factor 2, and Gli1/signal transducer and activator of transcription 3 axis. Evidence specific to various cancer types, including nasopharyngeal, lung, colorectal, gastric, hepatocellular, pancreatic, prostate cancer, and osteosarcoma, was critically evaluated. Translational strategies, such as combination therapy, nanodelivery systems, and machine learning-directed structural optimization, have been examined. Additionally, the challenges of low bioavailability, resistance to ferroptosis, and complex immunological responses are discussed. Preclinical evidence suggests that berberine exhibits significant epigenetic activity, including the inhibition of DNA methyltransferases (DNMT1/DNMT3), histone modifications (H3K9me3 via SETDB1, H3K27me3 via EZH2), and modulation of oncogenic/tumor-suppressor microRNAs (e.g., miR-21, miR-155). These actions enhance its ferroptotic effects and may synergistically increase the sensitivity of cancer cells to lipid peroxidation. Although primarily based on preclinical findings, these epigenetic mechanisms represent a crucial and underexplored aspect of berberine's anticancer potential.

See also the graphical abstract(Fig. 1).

## Introduction

Cancer cells frequently evade therapeutic interventions by neutralizing apoptotic pathways, leading to treatment failure and recurrence (Gu et al., 2025[21]). Most tumors overexpress anti-apoptotic proteins or functionally inactive p53, enabling survival during genotoxic and cytotoxic stress and neoplasmic multidrug resistance (Fulda, 2010[18]). This has further motivated the interest in non-apoptotic regulated forms of cell death, which can be utilized to destroy cancer cells resistant to apoptosis pharmacologically. Ferroptosis was initially characterized by Dixon et al. as an iron-dependent, non-apoptotic form of cell death induced by the RAS-selective lethal agent, erastin. It is distinguished by distinct morphological, genetic, and biochemical features compared to apoptosis, necrosis, and autophagy (Dixon et al., 2012[15]). This study defined ferroptosis as an iron-dependent death program that is closely connected to lipid peroxidation in cancer cells.

Subsequent mechanistic investigations have identified glutathione peroxidase 4 (GPX4) as a key negative modulator of ferroptosis in cancer cells (Li et al., 2025[40]). Yang et al. demonstrated that genetic knockdown or pharmacological inhibition of GPX4 induces selective ferroptotic cell death in human cancer cell lines. Overexpression of GPX4 prevents cells from undergoing ferroptosis in response to various inducers, and targeting GPX4 inhibits tumor growth in xenograft models (Yang et al., 2014[66]). Therefore, upstream glutathione synthesis through cystine importation is achieved by the cystine/glutamate antiporter system Xc-SLC7A11/SLC3A2. Jiang et al. established that SLC7A11 is directly suppressed by p53, reducing cystine uptake and glutathione levels, making cells susceptible to ferroptosis, and forcing SLC7A11 expression inhibited p53-induced tumor suppression in vivo (Jiang et al., 2015[29]). Antitumor immunity also involves ferroptosis: activated CD^8+^ T cells stimulate ferroptosis and tumor lipid peroxidation by interferon-g-dependent SLC7A11/SLC3A2 downregulation, and immunotherapy activity is inhibited by ferroptosis (Wang et al., 2019[62]).

Berberine, an isoquinoline alkaloid recognized for its anticancer properties, has been extensively documented to activate classical cell death pathways (Almatroodi et al., 2022[3]). Berberine increased p53 and Bax levels, decreased Bcl-2 levels, disrupted mitochondrial membrane potential, induced cytochrome c release, and activated caspase-3, leading to G2/M arrest and apoptosis in human gastric carcinoma SNU-5 cells (Lin et al., 2006[43]). Recent original research indicates that berberine directly engages ferroptosis-related mechanisms, resulting in similar outcomes. Yi et al. revealed that ferrous iron accumulation, glutathione depletion, and ROS-mediated ferroptosis in hepatic stellate cells through ferritin degradation are induced by berberine to resolve experimental liver fibrosis (Yi et al., 2021[68]). Wu et al. demonstrated that berberine repressed SLC7A11, SLC3A2, and GPX4, depleted glutathione, enhanced lipid ROS, and induced ferroptosis in nasopharyngeal carcinoma. Overexpression of GPX4 partially restores viability and minimizes metastasis in vivo (Wu et al., 2024[65]). Liao et al. also showed that berberine works in combination with ferroptosis inducers in non-small cell lung cancer by reducing SLC7A11, GPX4, and NRF2 in a p53-dependent manner, which increased tumor control in an allograft model (Liao et al., 2024[41]).

The novelty of the present study lies in its focused investigation of ferroptosis induced by berberine as an anticancer strategy, rather than exploring the broader aspects of ferroptosis biology or the extensive pharmacological properties of berberine. The current literature predominantly regards berberine as a peripheral component within a broad category of ferroptosis modulators or as an agent influencing apoptosis and cell cycle processes, without specifically addressing ferroptosis-related pathways. This study aimed to delineate the impact of berberine on the regulation of the system Xc^-^/GSH/GPX4 axis, iron metabolism, and lipid peroxidation in cancer. This review summarizes the findings specific to cancer types, both in vitro and in vivo, including their synergistic effects with ferroptosis inducers. Additionally, this study provides insights into translational challenges, such as combination therapies, nanodelivery systems, and biomarkers, situating berberine-mediated ferroptosis within the framework of experimentally substantiated and clinically oriented anticancer drug development.

## Ferroptosis: Core Molecular Machinery and Regulatory Axes

The execution of ferroptosis involves three biochemical pillars that are interdependent biochemical pillars: iron-catalyzed radical chemistry, peroxidation of select membrane phospholipids, and the breakdown of endogenous lipid-hydroperoxide detoxification mechanisms (Bayır et al., 2020[5]). This section outlines the original evidence of the experiments that form the basis of each pillar and the control axes that link the axes.

### System Xc^-^/GSH/GPX4 axis

The cystine/glutamate antiporter system Xc^-^ (SLC7A11/SLC3A2) is the canonical defense mechanism against ferroptosis, which stabilizes the supply of intracellular cysteine to maintain glutathione (GSH) production (Koppula et al., 2021[35]). Dixon et al. demonstrated that system Xc^-^, which depletes cysteine and collapses GSH pools, is inhibited by erastin and results in downstream lipid-hydroperoxide reductase GPX4 inactivation causing iron-dependent cell death, which is fully inhibited by the iron chelator deferoxamine (Dixon et al., 2012[15]). Yang et al. then made GPX4 the only enzyme that could reduce phospholipid hydroperoxides in biological membranes: genetic deletion or pharmacologic inhibition of GPX4 was lethal in lipid peroxidation in a wide range of cancer cell lines, and GPX4 overexpression was resistant to all 12 ferroptosis inducers; genetic GPX4 deletion or pharmacologic inhibition of GPX4 decreased tumor growth in xenograft models (Yang et al., 2014[66]). These results indicate that the Xc^-^GSH-GPX4 system is the best-validated pharmacological axis for controlling ferroptosis.

### Lipid substrates and ACSL4-dependent membrane remodeling

Membrane lipids are not equally competent in ferroptosis. Quantitative redox lipidomics and reverse genetics have revealed that ferroptosis depends on the selective oxidation of phosphatidylethanolamines (PEs) esterified with arachidonoyl (AA) or adrenoyl (AdA) fatty acyl chains, and exogenous PE-AA-OOH directly induces ferroptosis in cells deficient in PUFA-esterification (Kagan et al., 2017[31]). This channeling enzyme of these PUFAs into membrane phospholipids was also independently identified by Doll et al. by genome-wide CRISPR screening: acyl-CoA synthetase long-chain family member 4 (ACSL4) increases the concentration of ferroptotic oxidizable long-chain o6 PUFAs in membranes, and Gpx4-Acsl4 dual-knockout cells exhibited significant ferroptotic resistance (Doll et al., 2017[17]).

### Iron homeostasis and ferritinophagy

Labile iron that is present intracellularly catalyzes lipid peroxidation through Fenton chemistry and activates also iron-dependent lipoxygenases. Hou et al. showed that macroautophagy actively promotes ferroptosis and NCOA4-mediated lysosomal degradation of ferritin (ferritinophagy) (Chen et al., 2021[11]; Zeng et al., 2026[70]). Knockout or knockdown of Atg5 and Atg7 inhibits ferritin degradation, limits labile iron buildup, and inhibits erastin-triggered ferroptosis in fibroblasts and cancer cells (Hou et al., 2016[26]). Gao et al. also revealed that ferroptotic death and labile iron accumulation were selectively eliminated by NCOA4 knockdown, supporting ferritinophagy as a vital iron-delivery mechanism for executing ferroptosis (Gao et al., 2016[20]).

### GPX4-independent defense: the FSP1-CoQ_10_ axis

Two pioneering studies were conducted consecutively, serving as landmark studies. Beresuker et al. identified ferroptosis suppressor protein 1 (FSP1) using a synthetic lethal CRISPR-Cas9 screen (Cheu et al., 2023[13]). They found that the expression of FSP1 is positively correlated with resistance to ferroptosis in numerous cancer cell lines and lung cancer xenografts (Bersuker et al., 2019[6]). In independent studies, Doll et al. showed that myristoylated FSP1 localizes to the plasma membrane and acts as an NAD(P)H-dependent oxidoreductase that reacts with ubiquinol (reduced CoQ_10_), a lipophilic radical-trapping antioxidant. Pharmacological targeting of FSP1 cooperates with GPX4 inhibitors to induce ferroptosis in various cancers (Doll et al., 2019[16]).

These initial discoveries form a multi-layered regulatory framework: system Xc^-^/GSH/GPX4 and FSP1/CoQ10 are parallel defense barriers; ACSL4-induced PUFA-PE remodeling and NCOA4-meditated ferritinophagy are pro-ferroptotic catalysts; and labile iron is the focal catalyst. This network contains several pharmacologically targetable nodes that directly affect the mechanism of berberine-induced ferroptosis in cancer cells.

## Berberine in Oncology: From Classical Cell Death to Ferroptosis

The isoquinoline alkaloid berberine (BBR) is a natural product of Coptis chinensis and Berberis that has amassed a significant amount of first-order experimental data in a wide range of malignancies (Chauhan et al., 2024[7]). This section will follow the progression of classical apoptosis and autophagy research to the new ferroptosis paradigm.

### Apoptosis induction across cancer types

The first mechanistic data were obtained by Lin et al., who demonstrated that BBR causes G2/M arrest and caspase-3-dependent apoptosis through the upregulation of p53 and downregulation of Bcl-2 in human gastric carcinoma SNU-5 cells (Lin et al., 2006[43]). Patil et al. in their study exhibited that BBR induces mitochondrial apoptosis in breast cancer MCF-7 cells by releasing cytochrome c, activating caspase-9, and cleaving PARP in the cells in vitro with no effect on the normal MCF-12F cells (Patil et al., 2010[52]). Meeran et al. established that BBR being an apoptotic inducing agent activates ROS generation, mitochondrial depolarization and caspase-3 in PC-3 prostate cancer cells, but spares non-malignant RWPE-1 cells (Meeran et al., 2008[47]). Lin et al. established that in leukemia, BBR triggers caspase-3 in HL-60 and murine WEHI-3 cells through mitochondrial dysfunction caused by ROS/Ca2+ (Lin et al., 2006[42]). Mantena et al. also identified that BBR caused G1 arrest and p53/p21-mediated apoptosis in prostate LNCaP and DU145 cells in vitro and in nude mouse xenografts (Mantena et al., 2006[46]). Hsu et al also showed that BBR induces apoptosis through JNK/p38 MAPK and FasL on SW620 colon carcinoma cells (Hsu et al., 2007[27]).

### p53-dependent and cell-cycle regulatory mechanisms

Katiyar et al. established that BBR induced apoptosis in p53-positive A549 cells and p53-negative H1299 cells, and oral administration of BBR prevented A549 and H1299 xenografts from grow in p53- dependent athymic nude mice (Katiyar et al., 2009[34]). Choi et al. established the dependence of p53 in LNCaP prostate cancer xenografts over PC-3. In five epithelial cancer cell lines (Tca8113, HeLa, CNE2, MCF-7, and HT29) (Choi et al., 2009[14]), Li et al. observed uniform upregulation of BAX and downregulation of BCL-2 as a universal apoptotic process (Li et al., 2023[39]).

### Autophagy as a parallel death mechanism

Wang et al. showed that BBR activates AMPK /mTOR/ULK1-mediated autophagy and decreases tumor growth in U251 and U87 glioblastoma cells in vitro and in vivo (Wang et al., 2016[59]). Wang et al. demonstrated that BBR induces autophagy and apoptosis in hepatocellular carcinoma (HCC) HepG2 and MHCC97-L cells, and that BBR triggers cell death by inhibiting Atg5 (Wang et al., 2010[61]). La et al. showed that BBR increases GRP78 to cause selective autophagic death in HCT-116 and HepG2 cancer cells, but not in normal HL-7702 cells (La et al., 2017[36]).

### Combination therapy and sensitization

Yang et al. demonstrated that BBR can improve radiosensitivity in vitro and in an esophageal squamous cell carcinoma mouse xenograft model through the inhibition of HIF-1a/VEGF (Yang et al., 2013[67]). BBR increased the radiosensitivity of hepatoma by inhibiting the Nrf2 pathway in both Huh7/HepG2 and xenografts (Guo et al., 2019[22]). Liu et al. showed that BBR sensitizes cisplatin-resistant SKOV3 cells through the miR-21/PDCD4 axis in ovarian cancer (Liu et al., 2013[44]). Chen et al. demonstrated that BBR circumvents cisplatin resistance in A2780/DDP ovarian cells via miR-93/PTEN/AKT (Chen et al., 2015[9]). BBR also cooperated with doxorubicin in MCF-7 breast cancer cells and xenografts through the AMPK-HIF-1a-P-gp pathway (Pan et al., 2017[51]). Ming et al. demonstrated that in pancreatic PDAC cells, BBR suppresses mTORC1/ERK through AMPK and slows down the growth of MiaPaCa-2 xenografts by 70% (Ming et al., 2014[48]).

### Transition to ferroptosis

Yi et al. provided initial empirical evidence linking BBR to ferroptosis, demonstrating that BBR ameliorates liver fibrosis by inducing ferroptosis in hepatic stellate cells (HSCs). This process involves the accumulation of ferrous iron, reactive oxygen species (ROS)-mediated ferritin degradation, glutathione (GSH) depletion, and lipid peroxidation in mouse models of fibrosis induced by TAA and CCl4. Notably, these activities were reversed by ferrostatin-1 (Yi et al., 2021[68]). Zhu et al. demonstrated that in nasopharyngeal carcinoma (NPC), BBR provoked ferroptosis by suppressing the System Xc^-^/GSH/GPX4 axis: SLC7A11 and GPX4 were depressed, lipid ROS and Fe2+ accumulated, cristae of the mitochondrion fractured, and Fer-1/DFO inhibited BBR-induced mortality; BBR inhibited NPC lung metastasis in a mouse xenograft model (Wu et al., 2024[65]). In NSCLC, Liao et al. showed that BBR cooperates with a variety of ferroptosis inducers, which synergistically suppress SLC7A11, GPX4, and NRF2 with greater GSH depletion and MDA/ROS accumulation; the combination blocked tumor growth in mice allografts (depending on the presence of p53) (Liao et al., 2024[41]). In the most recent study, BBR induced ferroptosis-related energy metabolism disorder in HCT116 and CT26 cells (10-40 mM) and 80 mg/kg in vivo by suppressing the Gli1/STAT3 axis, promoting Fe2+, MDA, LDH, and inhibiting ATP and SOD, in colorectal cancer (Sun et al., 2026[56]). Li et al. also found that BBR sensitizes HCC cells to sorafenib by promoting SETDB1/NQO1/p53-mediated ferroptosis with lipid peroxidation alleviated by liproxstatin-1 and ferrostatin-1 (Li et al., 2025[38]).

In conclusion, original experimental research has consistently expanded the oncology profile of berberine beyond its traditional roles as a pro-apoptotic and autophagy-stimulating agent, revealing its capacity to directly interact with ferroptosis systems. The System Xc^-^/GSH/GPX4 axis, iron homeostasis, and p53-NRF2 signaling emerge as convergent nodes that are interconnected with the diverse death-promoting activities of berberine. These interactions provide a mechanistic rationale for the cancer-type-specific analyses presented in the subsequent sections.

## Epigenetic Regulation by Berberine in Cancer

### DNA methylation modulation

Berberine is a pharmacological regulator of DNA methylation by inhibiting major DNA methyltransferases, especially, DNMT1 and DNMT3B in cancerous cells. Berberine reduces the expression of DNMT1/DNMT3B in multiple myeloma U266 cells changing the hypomethylation of CpG islands in the TP53 promoter and the re-establishment of p53 signaling, a core of ferroptosis sensitivity (Qing et al., 2014[53]). Other Similar DNMT inhibition has been found in solid tumors where berberine decreases DNMT1/3 expression and changes promoter methylation of oncogenes and xenobiotic-metabolizing genes, which play a role in growth arrest and apoptosis. This data suggests that the tumor suppressor genes can be epigenetically reactivated by targeted promoter demethylation by berberine and thus offer an upstream mechanism that serves to synergize with the p53-SLC7A11 axis already involved in ferroptosis induced by berberine (Zhang et al., 2016[71]).

### Histone modification

Berberine also remodels histone in cancerous cells through histone methylation and acetylation. Berberine in an osteosarcoma model lowers the global levels of H3K27me3 and the histone methyltransferase EZH2, which is linked to reversal of the epithelial-mesenchymal transition (EMT), downregulation of vimentin/N-cadherin, upregulation of E-cadherin and reduced migration and invasion. These data prove that oncogenic Polycomb-mediated repression can be counteracted by berberine and, thus, transcriptional programs that support metastasis can be reprogrammed (Mishra et al., 2020[49]). Berberine is a chromatin-remodeling medication that inhibits the activity of total and class I/II/IV histone deacetylase-HDAC in human lung cancer A549 cells resulting in hyperacetylation of core histones, G1 cell-cycle arrest, and caspase-dependent apoptosis. Collectively, these results suggest that berberine is an epigenetic regulator of histone methyltransferases as well as HDACs, which offers another upstream process through which it can affect tumor suppressor and stress-response gene expression in terms of ferroptosis vulnerability (Kalaiarasi et al., 2016[32]).

### microRNA regulation

Berberine also has other effects in epigenetic regulation by regulating oncogenic and tumor- suppressor microRNAs within cancerous cells. Berberine is able to downregulate the expression of oncomiR miR-21 in human colon carcinoma HCT116 cells, which deregulates the expression of the oncomiR miR-21 downstream targets ITGb4 and tumor suppressor PDCD4; reconstruction of the miR-21-ITGb4-PDCD4 axis results in impaired cell survival, elevated apoptosis and caspase-3 activity (Lü et al., 2018[45]). Berberine equally inhibits miR-21, activates PDCD4, and significantly enhances cisplatin-mediated apoptosis, reversing chemoresistance by miR-21/PDCD4 interaction in cisplatin-resistant SKOV3 ovarian cancer cells. Collectively, these findings confirm that berberine has the potential to rearrange microRNA networks to reenable tumor-suppressive pathways, which is another regulatory level that can intersect with ferroptosis-related nodes, including p53 and stress-response genes (Liu et al., 2013[44]).

### Epigenetic-ferroptosis crosstalk

Growing evidence suggests that ferroptosis and epigenetic remodeling are mechanistically related to each other in cancer cells. The transcriptional output of the p53-SLC7A11-GPX4 axis and other pathways that regulate lipid-peroxidation is critically influenced by histone methyltransferases that include SETDB1 and EZH2, and promoter DNA methylation levels of the ferroptosis-regulatory genes. Ferroptosis is also further ingrained into the epigenetic network by non-coding RNAs since several ferroptosis-related lncRNAs and microRNAs have the ability to regulate the level of NRF2, GPX4, and SLC7A11 and, in doing so, dictate cell sensitivity to iron-dependent lipid peroxidation. In this context, the capacity of berberine to inhibit DNMTs, remodel histone methylation, and reprogram miRNAs indicates that its epigenetic and ferroptotic activities are both conceptually connected by the idea that the epigenetic and ferroptotic effects of the drug place tumor cells in a position to undergo ferroptotic death through deregulating p53 and disrupting antioxidant-defense genes (Wang et al., 2023[63]).

## Molecular Mechanisms of Berberine-Induced Ferroptosis

Ferroptosis is a regulated form of cell death that is dependent on iron and is characterized by lipid peroxidation. It has recently emerged as a potential vulnerability in oncology, offering opportunities for therapeutic exploitation of cancer cells (Chen et al., 2024[8]). Berberine (BBR) has been identified as a pleiotropic inducer of ferroptosis, disrupting antioxidant defenses, iron homeostasis, and mitochondrial bioenergetics in various cancer types (Cheng et al., 2025[12]).

The most thoroughly described process involves the inhibition of the System Xc^-^/GSH/GPX4 axis. Wu et al. first demonstrated the role of BBR in inducing ferroptosis in nasopharyngeal carcinoma (NPC) cells by dose-dependently suppressing SLC7A11, SLC3A2, and GPX4 mRNA and protein levels, leading to GSH depletion, ROS accumulation, and Fe2+ overload. They rescued cell death caused by BBR using ferrostatin-1 (Fer-1) and deferoxamine (DFO) (Wu et al., 2024[65]). This axis was also validated in prostate cancer DU145 and PC-3 cells, in which Fer-1, and not apoptosis or pyroptosis inhibitors, reversed the cytotoxicity of BBR, and SLC7A11 overexpression inhibited ferroptosis (Zou et al., 2025[72]). Alassiri et al. also showed that BBR induced ferroptosis in oral cancer KB-1 cells through the GPX4/STEAP3 pathway, and both targets bind to it strongly, which is supported by molecular docking (Alassiri, 2025[2]).

p53 is a tumor suppressor that acts as a key gatekeeper of ferroptosis sensitivity mediated by BBR (Kang et al., 2019[33]). Liao et al. demonstrated that BBR with erastin and RSL3 can co-downregulate SLC7A11, GPX4, and NRF2 in NSCLC cells, which was eliminated in p53-mutant cells (Liao et al., 2024[41]). In line with this, Li et al. established that BBR sensitizes hepatocellular carcinoma cells to SETDB1/NQO1/p53-mediated ferroptosis with deleterious mitochondrial dysfunction, which is reversible by liproxstatin-1 (Li et al., 2025[38]). BBR has also been shown to induce ferroptosis in osteosarcoma MG63 cells through the inter-reluctant STAT3/p53/SLC7A11 signaling node (Jianjun and Wenkui, 2025[30]).

Another vital mechanism is mitochondrial bioenergetic disruption. Mori et al. revealed that BBR suppresses the mitochondrial complex I in the gastrointestinal cells with cancer, leading to pathological accumulation of Fe2+, the increase of 4-HNE, and mitophagy mediating by Parkin/PINK1 under the influence of the BBR; and Fer-1, DFO, Z-VAD- FMK, and ATG5 knockdown partially reversed the cell death, which proved the combined ferroptosis-apoptosis-autophagy phenotype (Mori et al., 2023[50]). Sun et al. discovered a novel Gli1/STAT3-ferroptosis negative regulation (FNR) axis in colorectal cancer with direct BBR-Gli1 binding (K d = 0.652 mM) mediated by surface plasmon resonance; the inhibition of this axis caused collapse of ferroptosis-driven energy metabolism. The activation of the ferroptosis pathway via nanoparticle-encapsulated BBR also enhanced ferroptosis in CRC models compared to free BBR (Sun et al., 2026[56]). Finally, Yi et al. demonstrated that BBR removes activated hepatic stellate cells via ROS-mediated ferroptosis, which occurs because of redox perturbation of iron, reducing liver fibrosis in vivo (Yi et al., 2021[68]).

**Figure 2:**
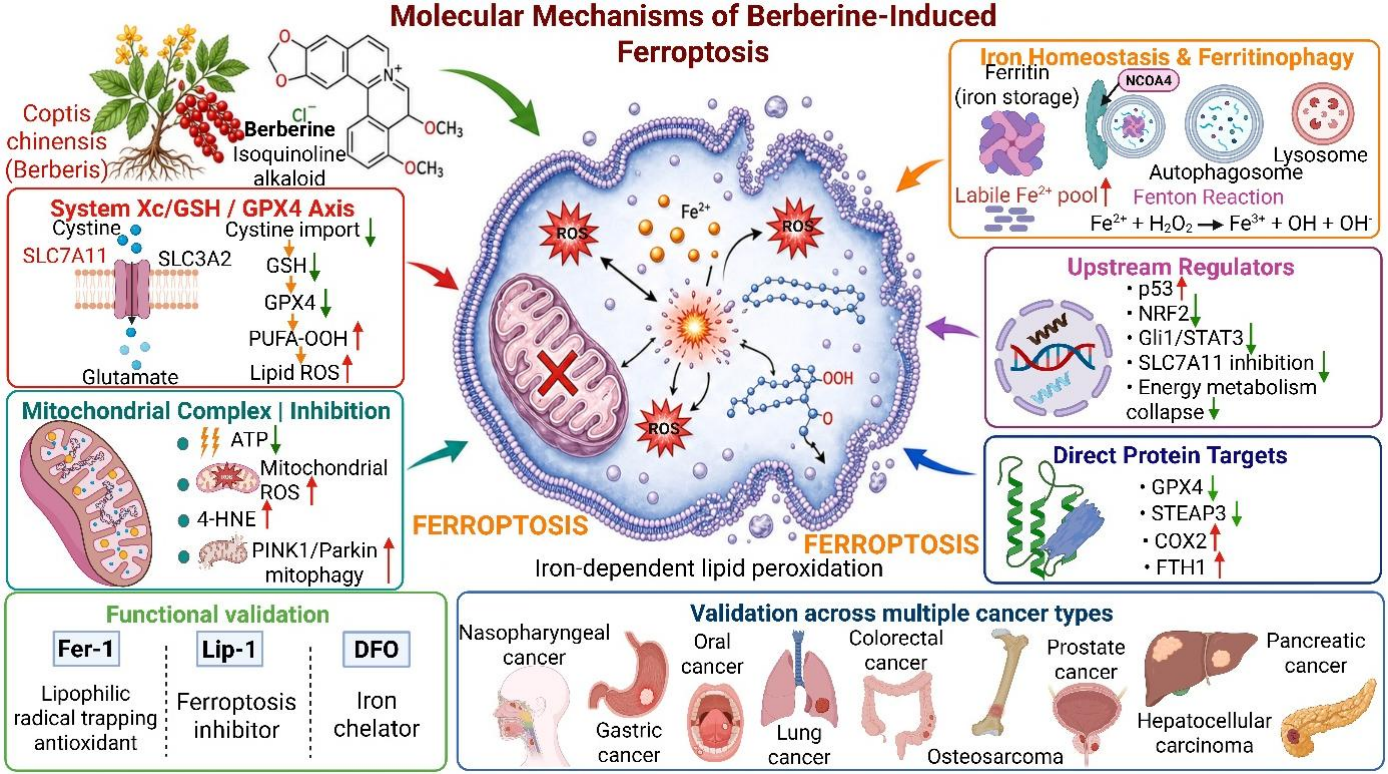
Molecular mechanisms of berberine-induced ferroptosis; created with BioRender.com

Collectively, these studies substantiate that BBR induces ferroptosis by disrupting the coordinated System Xc^-^/GSH/GPX4, stabilizing p53, inhibiting mitochondrial complex I in conjunction with mitophagy, and targeting novel pathways such as Gli1/STAT3-FNR and GPX4/STEAP3 pathways. This provides a mechanistic rationale for the applicability of BBR-based ferroptosis-sensitization strategies in various malignancies (Figure 2(Fig. 2)).

## Cancer Type-Specific Evidence: In Vitro and In Vivo Studies

Ferroptosis induced by berberine (BBR) has been documented in various solid tumors; however, the most comprehensive mechanistic insights have been elucidated in studies focusing on head and neck and thoracic tumors (Wu et al., 2024[65]). In the context of nasopharyngeal carcinoma (NPC), Wu et al. demonstrated that the inhibition of the System Xc^-^/GSH/GPX4 axis by BBR in S18 and 5-8F cells resulted in dose-dependent increases in reactive oxygen species (ROS), lipid peroxidation, and intracellular Fe2+. The restoration of cell viability by ferrostatin-1 (Fer-1) and deferoxamine (DFO) provides functional confirmation of ferroptotic cell death (Wu et al., 2024[65]). Extending this research to oral squamous cell carcinoma, Alassiri showed that BBR induced ferroptosis and inhibited the migration of KB-1 cells (IC_50_ ≈ 22 μM) by downregulating GPX4 and STEAP3 while upregulating ferroportin. Molecular docking studies have revealed a strong direct interaction between BBR and both targets (Alassiri, 2025[2]). Liao et al. found that in non-small cell lung cancer (NSCLC), BBR and canonical ferroptosis inducers (erastin, RSL3) synergistically depletes GSH and inhibits SLC7A11, GPX4, and NRF2 via a p53-dependent mechanism, but not in p53-mutant cells, and in a lung cancer allograft model, the combination outperformed either agent alone (Liao et al., 2024[41]).

In the gastrointestinal tract, BBR triggers ferroptotic mechanisms that differ but overlap in gastric, intestinal, and colorectal tumors (Hou et al., 2024[25]). Mori et al. demonstrated that BBR inhibits mitochondrial complex I in three gastrointestinal cancer cell lines (HGC-27, NUGC-4, and DLD-1) and induces pathological mitochondrial accumulation of Fe2+, increased levels of 4-hydroxynonenal (4-HNE), depletion of GSH, reduction of GPX4, and Parkin/PINK1-mediated mitophagy, culminating in cell death. This process is partially inhibited by Fer-1, DFO, Z-VAD-FMK, and ATG5 (Mori et al., 2023[50]). Specifically, in the context of gastric cancer, Yi et al. discovered that ferroptosis inhibitors suppress BBR-induced cell death, and BBR-induced autophagy enhances the occurrence of ferroptosis, indicating a crosstalk between autophagy and ferroptosis in gastric carcinoma cells. One more evidence of redox-dependent non-apoptotic death programs in cancer type-specific evidence was demonstrated by Complementary network pharmacology and experimental validation in AGS stomach carcinoma cells by Han et al. that BBR increases intracellular ROS and malondialdehyde (MDA) and reduces Nrf2/ HO-1 defenses (Han et al., 2025[23]), and by Wang et al. further demonstrates that BBR triggers mitochondrial membrane potential collapse, ATP decrease, and upregulation of p53 and AMPK in stomach carcinoma Sun et al. mapped a new Gli1/STAT3-ferroptosis negative regulation (FNR) axis (which was identified in colorectal cancer (CRC)) in which surface plasmon resonance confirmed BBR-binding on Gli1 and subsequent ferroptosis-induced energy metabolism blockage and considerable tumor growth inhibition in vivo (Wang et al., 2024[60]). Shen et al. demonstrated that PEG-PLGA nanoparticles loaded with berberine (NPBer) increased the intratumor accumulation of berberine in HCT116 xenograft mice. Transcriptomic profiling of HCT116 xenografts and PEG-PLGA nanoparticles loaded with berberine (NPBer) revealed enhanced activation of ferroptosis and mitophagy pathways and tumor suppression compared to free BBR (Shen et al., 2024[54]).

Ferroptosis induced by BBR has been confirmed by functional rescue experiments in genitourinary and musculoskeletal malignancies (Sun et al., 2026[56]). The study conducted by Zhu et al. on prostate cancer DU145 and PC-3 cell lines demonstrated that Fer-1, as opposed to apoptosis or pyroptosis inhibitors, effectively reversed the loss of viability induced by BBR. The study further indicated that BBR decreased the levels of GSH and cysteine, increased lipid ROS and Fe2+, inhibited SLC7A11 and GPX4, and upregulated COX2 and FTH1. Notably, SLC7A11 overexpression reversed all ferroptosis markers, whereas BBR led to the depletion of GSH and cysteine. In a complementary network pharmacology study, Zou et al. identified TYMS and AR as additional ferroptosis-related targets regulated by BBR in prostate cancer, thereby broadening the potential biomarker space for patient stratification (Zou et al., 2025[72]). Ji and Qiu identified a STAT3/p53/SLC7A11 signaling cascade in osteosarcoma MG63 cells in which BBR induced ferroptosis; BBR inhibited STAT3, deregulated p53, and inhibited SLC7A11, and p53 knockdown or STAT3 overexpression each reverted lipid ROS, Fe2+, MDA, and GSH depletion ferroptotic biomarkers (Jianjun and Wenkui, 2025[30]). To accelerate translational development, He et al. developed B1, a derivative of berberine, which employs machine learning-aided design to attract the E3 ligase NEDD4L to ubiquitinate and degrade stearoyl-CoA desaturase (SCD) to induce saturated lipid buildup, mitochondrial damage, and ferroptosis mediated by GPX4 in various osteosarcoma cell lines, and showed better in vivo efficacy compared to native BBR in subcutaneous and orthotopic xenograft models (He et al., 2025[24]).

**Figure 3:**
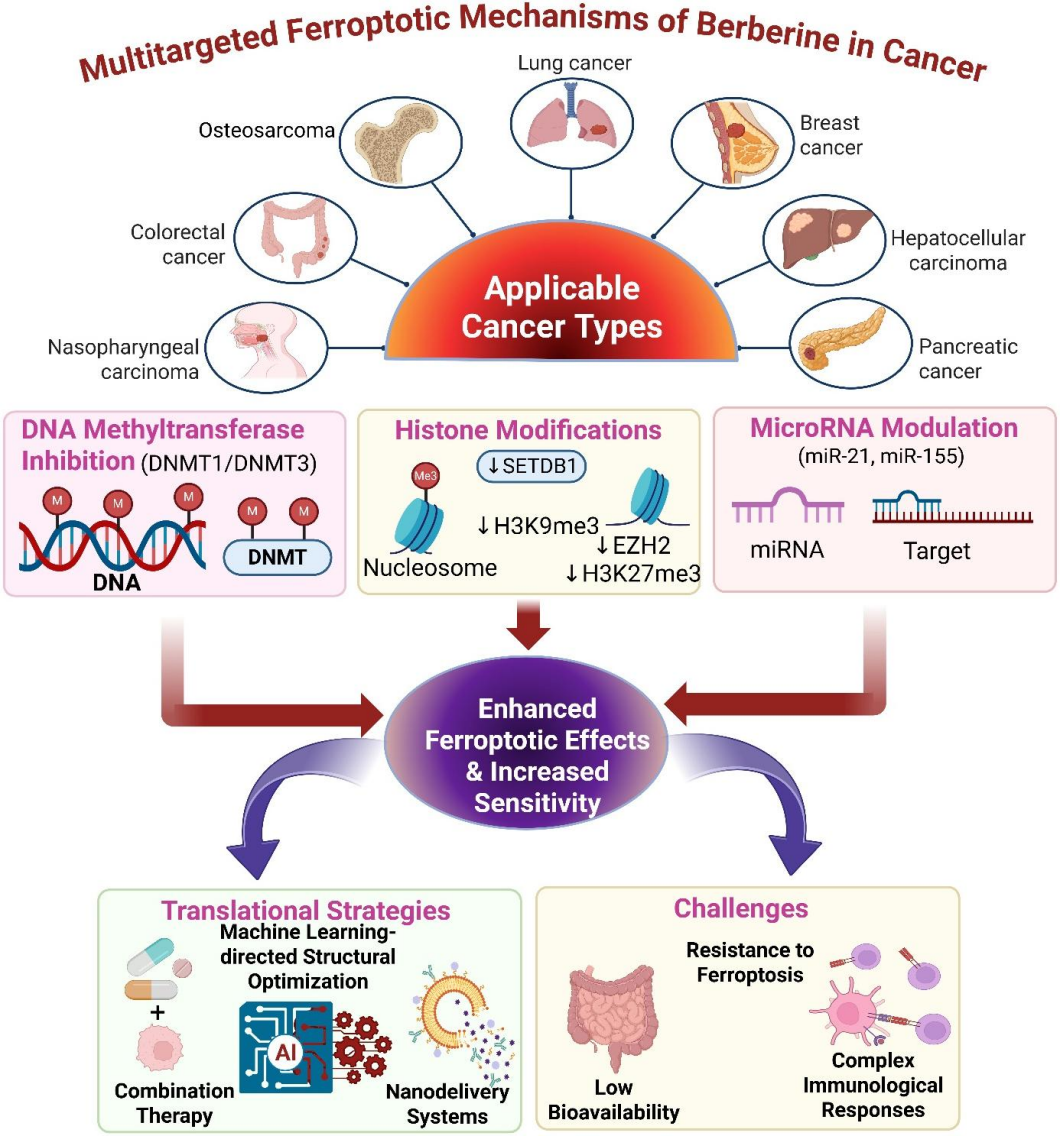
Multitargeted ferroptotic mechanisms of berberine in cancer; created with BioRender.com

Hepatobiliary and pancreatic cancers also support the versatility of ferroptosis-inactivating BBR and its ability to eliminate therapeutic drug resistance. Li et al. demonstrated that berberine (BBR) enhances the tolerance of hepatocellular carcinoma (HCC) cells to sorafenib via ferroptosis mediated by the SETDB1/NQO1/p53 pathway. This effect is amplified when BBR treatment is combined, exacerbating mitochondrial dysfunction, reactive oxygen species (ROS) accumulation, and lipid peroxidation, thereby positioning BBR as a ferroptosis-directed chemosensitizing adjuvant (Li et al., 2025[38]). Consistent with the potential of BBR to reprogram hepatic redox biology, Yi et al. found that in activated hepatic stellate cells, BBR stimulates ferrous redox cycling, promotes ferritin degradation via the ubiquitin-proteasome complex, and induces ROS-mediated ferroptosis, thereby ameliorating liver fibrosis in TAA/CCl4 mouse models of liver fibrosis. This antifibrotic activity is neutralized by Fer-1 (Yi et al., 2021[68]). Wang et al. developed carrier-free berberine-artesunate self-assembly nanoparticles (BBR-ART NPs) for the treatment of pancreatic cancer, specifically targeting ferroptosis in PANC-1 cells by upregulating HO-1 and downregulating GPX4 and xCT. In vivo, BBR-ART NPs exhibited the highest xenograft tumor-inhibitory effect among all treatment types. This effect was significantly reduced by co-administration of Fer-1, indicating a ferroptosis-dominated mechanism with acceptable systemic safety. Collectively, these 15 studies demonstrate that BBR activates context-dependent ferroptotic nodes, such as System Xc^-^/GPX4, p53/SLC7A11, STAT3/p53/SLC7A11, Gli1/STAT3-FNR, SETDB1/NQO1/p53, mitochondrial complex I, and NEDD4L-SCD, across various malignancies, all of which have been validated in vivo (Wang et al., 2024[64]) (Figure 3(Fig. 3); Table 1(Tab. 1); References in Table 1: Alassiri, 2025[2]; Han et al., 2025[23]; He et al., 2025[24]; Jianjun and Wenkui, 2025[30]; Li et al., 2025[38]; Liao et al., 2024[41]; Mori et al., 2023[50]; Shen et al., 2024[54]; Sun et al., 2026[56]; Wang et al., 2024[60]; Wang et al., 2024[64]; Wu et al., 2024[65]; Yi et al., 2021[68]; Zou et al., 2025[72]).

## Translational Perspectives: Combination Strategies, Nanodelivery, and Overcoming Resistance

While berberine (BBR)-induced ferroptosis has demonstrated significant preclinical efficacy across a diverse array of malignancies, its clinical application has been constrained by the compound's exceedingly low oral bioavailability, which is less than 1 percent in rats. This limitation is attributed to its low aqueous solubility, extensive first-pass metabolism, and active intestinal efflux mediated by the P-g-glycoprotein (P-gp) pump (Chen et al., 2011[10]). These challenges in drug delivery necessitate the development of advanced drug delivery systems, strategic combination therapies, and structural modifications to achieve a therapeutically significant induction of ferroptosis at the tumor site.

 The most clinically available translational method is combination therapy. In non-small cell lung cancer (NSCLC), Liao et al. reported that BBR forms additive synergies with canonical ferroptosis inducers (erastin, RSL3, and sulfasalazine) to co-suppress the SLC7A11/GPX4/NRF2 antioxidant axis, which is much more effective in tumor inhibition when combined with either of the two agents, making it highly important to consider patient stratification by TP53 genomic status (Liao et al., 2024[41]). Li et al. demonstrated that BBR sensitizes liver cancer cells to sorafenib through the induction of SETDB1/NQO1/p53-dependent ferroptosis and genomic instability, impairing mitochondrial dysfunction, reactive oxygen species (ROS) generation, and lipid peroxidation levels beyond those possible with sorafenib alone. Ferroptosis inhibitors (liproxstatin-1 and ferrostatin-1) neutralized this synergistic cytotoxicity, making ferroptosis the dominant cell death modality and positioning BBR as a ferroptosis-directed chemosensitizing adjuvant capable of overcoming acquired kinase-inhibitor resistance (Li et al., 2025[38]).

Nanodelivery platforms provide a parallel solution to the bioavailability deficiency of BBR, while simultaneously increasing tumor-selective ferroptosis. Shen et al. showed that PEG-PLGA nanoparticle-encapsulated berberine (NPBer) had high cellular uptake and intratumoral retention in HCT116 colorectal cancer xenografts, and RNA-seq transcriptomic profiling revealed an increased activation of ferroptosis and mitophagy pathways; in vivo, NPBer had a significant tumor suppressive effect compared to free BBR with no organ toxicity (Shen et al., 2024[54]). Taking advantage of carrier-free nanotechnology, Wang et al. established self-assembling nanoparticles consisting of 100 percent BBR and 100 percent artesunate (BBR-ART NPs) against pancreatic cancer. BBR-ART NPs (~80% inhibition rate) accumulated in PANC-1 xenografts and synergistically triggered iron-dependent ferroptosis by downregulating GPX4 and upregulating heme oxygenase-1 (HO-1). The extensive tumor suppression induced by BBR-ART NPs (approximately 80 % inhibition rate) was attenuated by the addition of ferrostatin-1, indicating a ferroptosis-dominated mechanism with acceptable systemic safety. These platforms not only solve the problem of inadequate exposure to the tumor but also the issue of multi-node redox/iron perturbation to overcome exposure to intrinsic stress-buffering mechanisms (Wang et al., 2024[64]).

In addition to native BBR, rational structural optimization enhances translational viability. He et al. employed a machine learning-guided design to develop B1, a 9-O-substituted derivative of berberine. This compound functions as a molecular glue, recruiting the E3 ligase NEDD4L to ubiquitinate and degrade stearoyl-CoA desaturase (SCD), thereby inducing saturated lipid accumulation, mitochondrial damage, and GPX4-associated ferroptosis in osteosarcoma cells. Notably, B1 exhibits significantly improved in vivo activity compared to that of native BBR (He et al., 2025[24]). Collectively, these translational advancements, including combination-based chemosensitization, nano-enabled targeted delivery, and AI-guided structural optimization, address the primary challenges of low bioavailability, insufficient tumor accumulation, and context-dependent resistance, bringing BBR-mediated ferroptosis closer to clinical evaluation.

## Challenges

While the preceding paragraphs highlight berberine (BBR) as an effective multi-target ferroptosis modulator with demonstrated preclinical efficacy, numerous formidable challenges must be addressed before it can be translated into clinical practice.

### Pharmacokinetic limitations

The primary challenge remains the notably low oral bioavailability of BBR, which is less than 1 percent in rodents and has a Cmax of 0.4 ng/mL in humans following a 400 mg oral dose. This low bioavailability is attributed to extensive first-pass metabolism by UDP-glucuronosyltransferases and active efflux mediated by P-glycoprotein (P-gp) and organic cation transporters. Although nanodelivery systems, such as PEG-PLGA nanoparticles and carrier-free self-assemblies, can significantly enhance tumor accumulation in xenograft models, none have been identified as clinical candidates for delivering BBR-mediated ferroptosis (Sun et al., 2024[55]). The hurdles of scalable manufacturing, long-term stability of nanoparticles, and regulatory approval of such formulations are yet to be overcome.

### Context-dependent effects and dual-edged effects

The interaction between ferroptosis and berberine (BBR) is unidirectional. BBR serves as a potent inducer of ferroptosis in cancer cells by inhibiting the SLC7A11/GPX4 axis. Conversely, BBR promotes ferroptosis in non-malignant cells through the activation of NRF2 and sustains ferroptosis in high-glucose-stimulated pancreatic β-cells by enhancing GPX4 expression levels. This bilaterality, while therapeutically beneficial, should tumor selectivity be achieved, poses the question of great importance of whether systemic administration of BBR may unwittingly protect tumor cells in some microenvironmental conditions, especially in the presence of NRF2 constitutive activity (Tavakoli et al., 2025[57]; Wang et al., 2024[64]). This will be solved with the help of extensive pharmacodynamic experiments on genetically characterized tumor subtypes.

### Ferroptosis resistance mechanisms

The efficacy of BBR may be compromised by various ferroptosis defense mechanisms employed by cancer cells, which operate through several pathways that are independent of GPX4 (Lei et al., 2022[37]). These include glutathione-independent lipid radical-trapping capacity facilitated by the FSP1/CoQ10 axis, DHODH/CoQH2 mitochondrial pathway, and GCH1/BH4 axis. In addition, MBOA1/2/2 upregulation by sex hormone receptors, cholesterol-mediated GPX4 stabilization through the SLC38A9-mTOR axis, and Wnt/β-catenin-based GPX4 transcription are new resistance nodes to which the existing repertoire of BBR does not directly respond (Jiang et al., 2024[28]). Future studies should systematically assess the ability of BBR to co-target these parallel defense systems or rational combinations with FSP1 inhibitors (e.g., iFSP1) or DHODH inhibitors (e.g., brequinar).

### Immunological complexity

The therapeutic mechanism of ferroptosis operates within a narrow immunological scope. Ferroptotic tumor cells release damage-associated molecular patterns (DAMPs), such as HMGB1, calreticulin, and ATP, which facilitate dendritic cell maturation and CD8+ T cell infiltration (Bathaei et al., 2025[4]). However, an excess of lipid peroxidation products, including 4-HNE and oxidized phospholipids, may counteract these effects by inhibiting dendritic cell activity and rendering effector T cells more susceptible to ferroptosis via the oxidation of membrane lipids. IFN-γ released by CD8+ T cells suppresses tumor SLC7A11 to induce ferroptosis but also enhances PD-L1, resulting in an anti-immune response (Yu et al., 2025[69]). Whether BBR-induced ferroptosis occurs within the constructive immunogenic window or is tilted rather than immune suppression is completely uninvestigated and forms a knowledge gap of paramount importance when used with immune checkpoint inhibitors.

### Biomarker and patient stratification gaps

The dependence of BBR synergy on p53 in conjunction with ferroptotic inducers in non-small cell lung cancer (NSCLC) underscores the importance of molecular stratification. Patients with gain-of-function mutations in TP53, alterations in the KEAP1/NRF2 signaling pathway, or elevated levels of FSP1/DHODH are likely to exhibit inherent resistance to BBR-induced ferroptosis (Adzavon et al., 2024[1]). There are no proven circulating or tissue-based biomarkers to predict ferroptosis sensitivity in the clinical environment; immunohistochemistry GPX4 and ACSL4, and serum lipid peroxidation markers (MDA, 4-HNE) are currently being proposed, awaiting prospective cohort validation (Wahida and Conrad, 2025[58]).

### Drug interaction concerns

BBR is a potent inhibitor of CYP3A4 and CYP2D6, both of which are enzymes responsible for the metabolism of approximately 60% of clinical agents at clinically relevant dosages (≥ 900 mg/day) (Gan et al., 2025[19]; He et al., 2025[24]). This poses a significant risk of pharmacokinetic drug-drug interactions when BBR is used with chemotherapeutic agents, targeted therapies, or immunotherapeutics. Before any combination regimen can be implemented, a thorough phase I pharmacokinetic-pharmacodynamic investigation is needed.

### Epigenetic off-target concerns

An additional layer of complexity in clinical translation is attributed to the epigenetic activity of berberine. Though the DNMT and HDAC modulation could restore the tumor suppressor gene expression, non-specific epigenetic remodeling can lead to unrestrained global gene expression profile, such as immune-regulatory and growth factors. The investigation would involve bisulfite sequencing and histone modification profiling of whole genome on BBR treated cancer models in future to establish the safe range of epigenetic modifications.

## Conclusion and Future Perspective

Berberine has emerged as a notable multitarget ferroptosis modulator, capable of disrupting intracellular redox homeostasis by inhibiting the SLC7A11/GPX4 antioxidant axis. It further disrupts iron metabolism through ferritinophagy and stimulation of heme oxygenase-1, thereby amplifying lethal lipid peroxidation across a broad spectrum of malignancies, including lung, liver, breast, pancreatic, colorectal, prostate, and nasopharyngeal cancers. In comparison to typical single-target ferroptosis inducers, berberine chronically interacts with upstream regulators, including NRF2, p53, BECN1, and ACSL4, thus affecting the redox defense network at more than one node and eliminating the possibility of adaptive resistance to any of the compensatory mechanisms. The distinctive mechanistic signature of isoquinoline alkaloids positions them as sophisticated metabolic regulators rather than merely cytotoxic agents. This characteristic enables the exploitation of the unique biochemical vulnerabilities of tumor cells that have developed resistance to conventional therapies. This underscores the significant potential of plant-based phytochemicals to redefine the current oncology paradigm.

The translational pathway from the bench to the bedside remains fraught with challenges. The poor oral bioavailability of berberine, its dual role as both an inducer of ferroptosis in tumor cells and a suppressor in normal tissues, the existence of GPX4-independent resistance mechanisms such as FSP1/CoQ10 and DHODH/CoQH2, the unknown immunological effects of berberine-induced ferroptosis within the tumor microenvironment, and the high likelihood of ferroptotic drug interactions with cytochrome P450 necessitate extensive research before clinical application. Recent investigations have focused on next-generation nanodelivery systems, carrier-free self-assemblies, and rational combinations of berberine with canonical ferroptosis inducers or conventional chemotherapeutics. These approaches have demonstrated synergy, the capacity to circumvent adaptive resistance networks, and enhanced therapeutic efficacy in preclinical models. In addition to having direct ferroptotic effects, the ability of berberine to remodel the cancer epigenome (by inhibiting DNMT, suppressing histone methyltransferase and miRNA regulation) provides another and complementary mechanism to sensitize resistant tumor cell and must be directly included in future mechanistic and translational studies.

To further achieve the clinical potential of ferroptosis mediated by berberine, the future will see the need to adopt a multidisciplinary approach based on precision oncology. The most pertinent future research should focus on identifying potent predictive biomarkers, such as lipidomic profiles, TP53 mutational status, and ferroptosis defense gene expression signatures, to rank patient groups that would respond best to this type of metabolic intervention. Concurrently, the structural optimization of the native berberine scaffold, facilitated by AI-driven computational modeling, alongside a thorough evaluation of its immunological effects in immunocompetent tumor model systems and initial human pharmacokinetic studies of nanoformulated berberine with ferroptosis-specific pharmacodynamic outcomes, will be pivotal in transforming berberine into a contemporary ferroptosis-targeted oncotherapeutic, as opposed to a traditional herbal remedy.

## Declaration

### Conflict of interest

The authors declare that they have no competing interests.

### Funding

None.

### Artificial Intelligence (AI) - assisted technology

The authors have not used any artificial intelligence (AI) assisted technology including large language models (LLMs), chatbots or AI-based image generators for the preparation of this manuscript. The scientific data have not been generated using any AI tool, and images, references, results interpretation and modifications to the scientific conclusions have been performed manually. All the figures in this article were generated with BioRender (BioRender.com) via a licensed, academic subscription. Any and all scientific content, textual writing, data interpretation and conclusions are solely the responsibility of the authors.

### Availability of data and materials

Not applicable.

### Consent for publication

Not applicable.

### Ethics approval and consent to participate

Not applicable.

### Author's contribution

Neetu Agrawal, Gaurav Gupta: Investigation, Writing-original draft & Formal analysis; Surya Nath Pandey, A Rekha: Visualization; Mano Priya Vijayan, Dinesh Kumar Chellappan: Formal analysis; Keshav Raj Paudal, Sachin Kumar Singh: Resources, Investigation & Conceptualization; Kamal Dua: Supervision, Investigation & Conceptualization.

## Figures and Tables

**Table 1:**
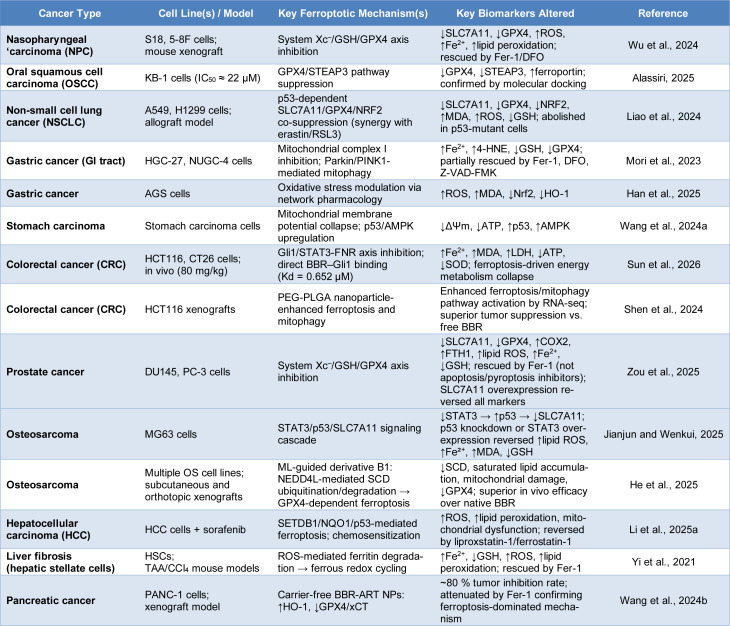
Summary of cancer type-specific evidence for berberine (BBR)-induced ferroptosis: in vitro and in vivo findings

**Figure 1:**
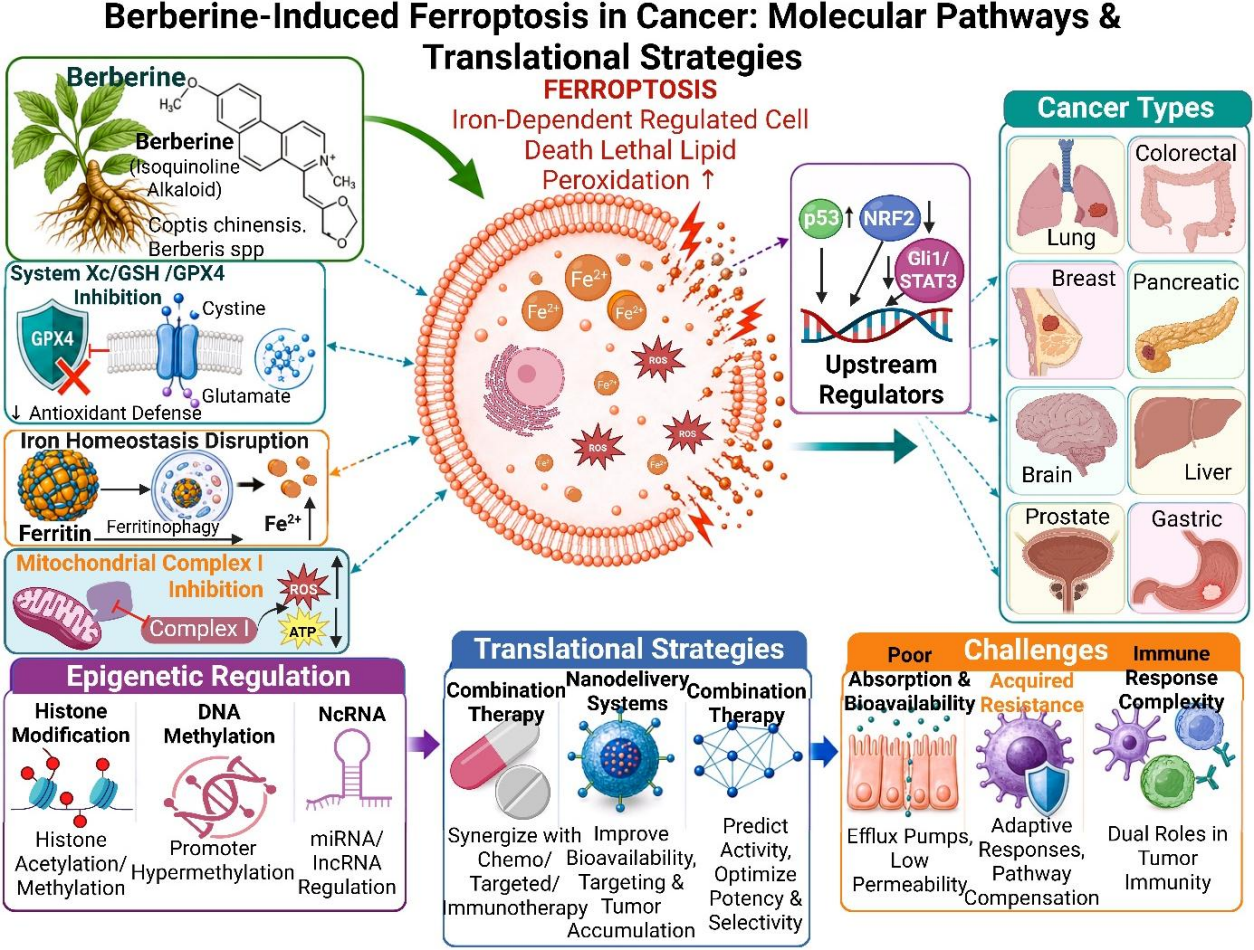
Graphical abstract
